# Menaquinone 4 increases plasma lipid levels in hypercholesterolemic mice

**DOI:** 10.1038/s41598-021-82724-0

**Published:** 2021-02-04

**Authors:** Jonna Weisell, Anna-Kaisa Ruotsalainen, Juha Näpänkangas, Matti Jauhiainen, Jaana Rysä

**Affiliations:** 1 School of Pharmacy, University of Eastern Finland, POB 1627, 70211 Kuopio, Finland; 2 A.I. Virtanen Institute for Molecular Sciences, University of Eastern Finland, Kuopio, Finland; 3 Department of Pathology, University of Oulu, Oulu University Hospital, Oulu, Finland; 4 Minerva Foundation Institute for Medical Research, Helsinki, Finland

**Keywords:** Pharmacology, Cardiovascular biology

## Abstract

In calcific aortic valve disease (CAVD) progressive valvular calcification causes aortic valve dysfunction. CAVD has several risk factors such as age and dyslipidemia. Vitamin K was shown to inhibit vascular calcification in mice and valvular calcification in patients with CAVD. We studied the effect of menaquinone 4 (MK4/vitamin K2) on valvular calcification in the hypercholesterolemic mouse model of CAVD. *LDLr*^*−/−*^*ApoB*^*100/100*^ male mice were fed with a Western diet for 5 months, with (n = 10) or without (n = 10) added 0.2 mg/g MK4. Body weight gain was followed weekly. Morphology of aortic valves and liver was assessed with immunohistochemistry. Plasma cholesterol levels and cytokines from hepatic tissue were assessed in the end of the study. Hepatic gene expression of lipid metabolism regulating genes were assessed after 18 h diet. MK4 exacerbated the lipoprotein lipid profile without affecting aortic valve morphology in hypercholesterolemic *LDLr*^*−/−*^* ApoB*^*100/100*^ mice. The MK4-containing WD diet increased plasma levels of LDL and triglycerides, hepatic steatosis, and mRNA expression of genes required for triglyceride and cholesterol synthesis. MK4 diminished levels of several cytokines and chemokines in liver, including IL-6, TNFα and MCP1, as measured by hepatic cytokine array. Consequently, MK4 may exert non-beneficial effects on circulating lipid levels, especially in hypercholesterolemic individuals.

## Introduction

Calcific aortic valve disease (CAVD) is the most prevalent valvular disease in the Western world^1^. The main features of CAVD pathogenesis are lipid accumulation, inflammation, fibrosis and calcification^1^. These changes cause thickening of the valves and eventually valvular dysfunction, leading to obstruction of blood flow. CAVD has several risk factors such as age, bicuspid aortic valve, male gender, hypertension, diabetes mellitus, and metabolic syndrome including dyslipidaemia. Main features of CAVD pathogenesis include lipid accumulation, inflammation, fibrosis and calcification. Low-density lipoprotein (LDL) and lipoprotein(a) levels have been shown to be causally associated with aortic stenosis^2,3^. Patients with familial hypercholesterolemia (FH) displayed a 7.9 -fold higher risk for CAVD^4^.

The efficacy of pharmacological therapies in the treatment of CAVD is unimpressive^1^. Recently, lipoprotein (a) and vitamin K targeted therapies have been studied in the treatment of CAVD^1^. Vitamin K1 and K2 (also known as menaquinones) belong to the lipid soluble vitamin K family^5^. Menaquinone 4 (MK4) and MK7 have been the most extensively studied members of the vitamin K2 family with respect to their clinical use. MK4 is also metabolised from vitamin K1. Vitamin K dependent Matrix Gla protein was reported to inhibit vascular calcification in mice; vitamin K1 and MK4 have been shown to attenuate warfarin-induced calcification in mice^6^ and the progression of CAVD in a proof-of-concept randomized controlled trial^7^.

As vitamin K has shown beneficial effects in the treatment of CAVD, we evaluated the effect of MK4 on the progression of CAVD in hypercholesterolemic transgenic mice expressing ApoB100 but knockout for the low‐density lipoprotein receptor (LDLr) (*LDLr*^*−/−*^*ApoB*^*100/100*^ mice). We demonstrate that MK4 increased plasma lipid levels and disturbed hepatic steatosis without affecting of CAVD in *LDLr*^*−/−*^*ApoB*^*100/100*^ mice.

## Results

### MK4 does not cause histological changes in aortic valves

**Figure 1:**
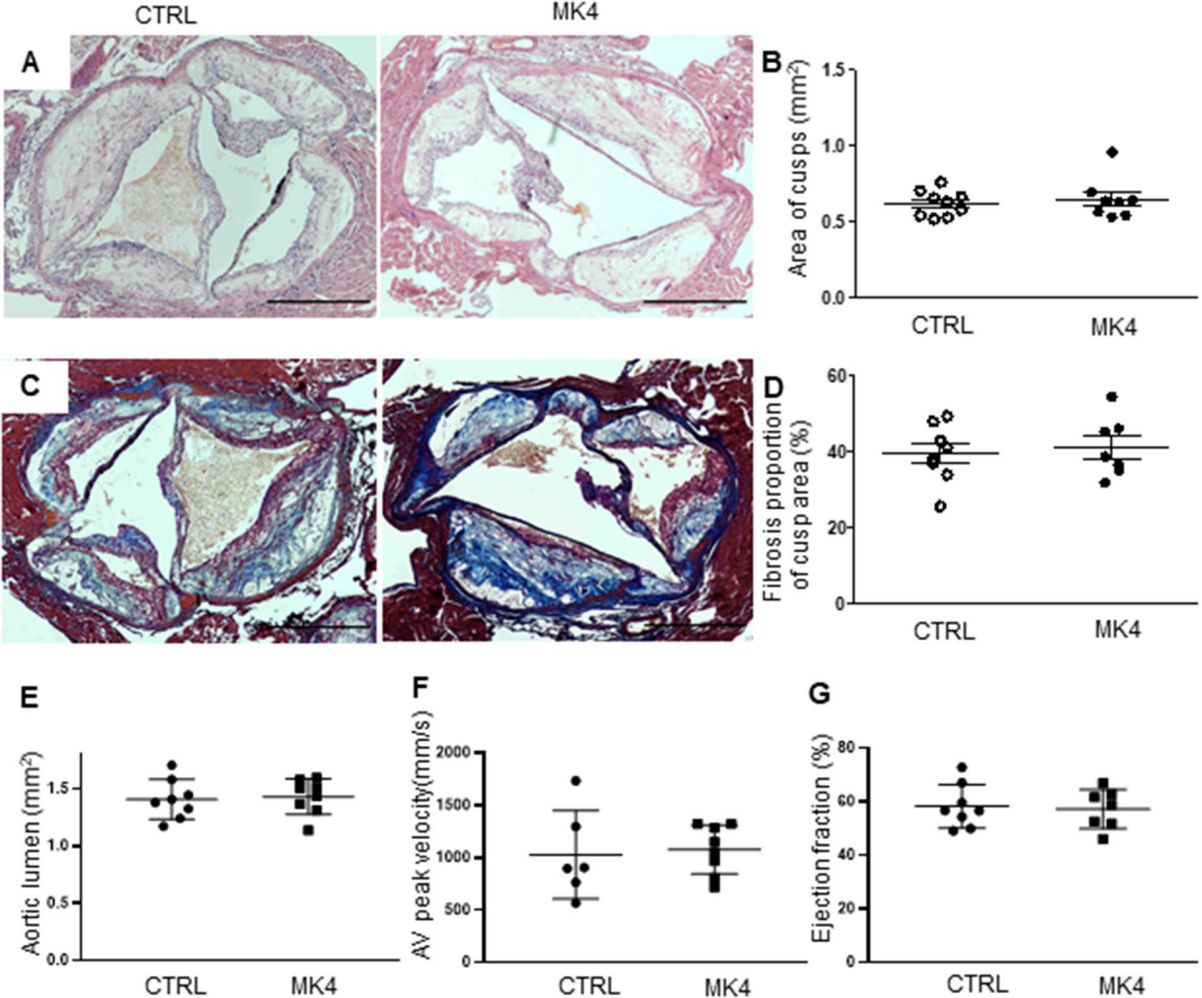
Effect of menaquinone 4 on aortic valve morphology in *LDLr*^*−/−*^*ApoB*^*100/100*^ mice. (**A**) Representative images of hematoxylin–eosin staining and (**B**) quantification of aortic cusp area. (**C**) Representative images of aortic valves with Masson trichrome staining for fibrosis, and (**D**) quantification of fibrosis in the aortic valves. Scale bars 500 µm. Mice heart functionality was assessed by echocardiography: quantification of (**E**) aortic valve peak velocity, (**F**) aortic lumen diameter, and (**G**) ejection fraction. CTRL (n = 6–8), *LDLr*^*−/−*^*ApoB*^*100/100*^ mice on Western diet (WD); MK4 (n = 7–8), *LDLr*^*−/−*^*ApoB*^*100/100*^ mice on WD with menaquinone 4. Student t-test was used for statistical analysis.

*LDLr*^*−/−*^*ApoB*^*100/100*^ mice were fed for 5 months with the WD which was supplemented with MK4; control mice received plain WD. This model mimics the structural and functional features of CAVD^8^. MK4 supplementation increased the plasma MK4 levels when compared to controls (*82.5* ± 57.7 *vs.* 5.2 ± 2.3 ng/ml*, p* < 0.05, respectively). We performed histological analyses to evaluate aortic valve leaflet morphology, calcification, and fibrosis. There were no changes in the aortic valve area between the control and MK4 fed mice (Fig. 1A–B) and the proportion of macrophages and calcification was similar between the study groups as assessed by MAC-3 immunohistochemistry and Alizarin red, respectively (Supplementary Fig. 1A–D). In addition, the amount of fibrosis in aortic valves was similar in both study groups (Fig. 1C–D) as determined with Masson trichrome staining. We also assessed mice heart functionality with echocardiography and aortic lumen diameter, peak jet velocity and ejection fraction were similar in both groups (Fig. 1E–G). In addition, we determined atheroma plaque area and calcification from atheroma plaques with Alizarin red staining. No changes were detected between the study groups either in the atheroma plaque area or calcification (Supplementary Fig. 1E–F). We also assessed plasma alkaline phosphatase (ALP) levels as ALP has been associated with vascular calcification^9^. A slight but not significant increase in plasma ALP levels were detected in MK4 fed mice when compared to control group (Supplementary Fig. 1G).

### MK4 increases plasma lipid levels and disturbed lipoprotein profiles

**Figure 2:**
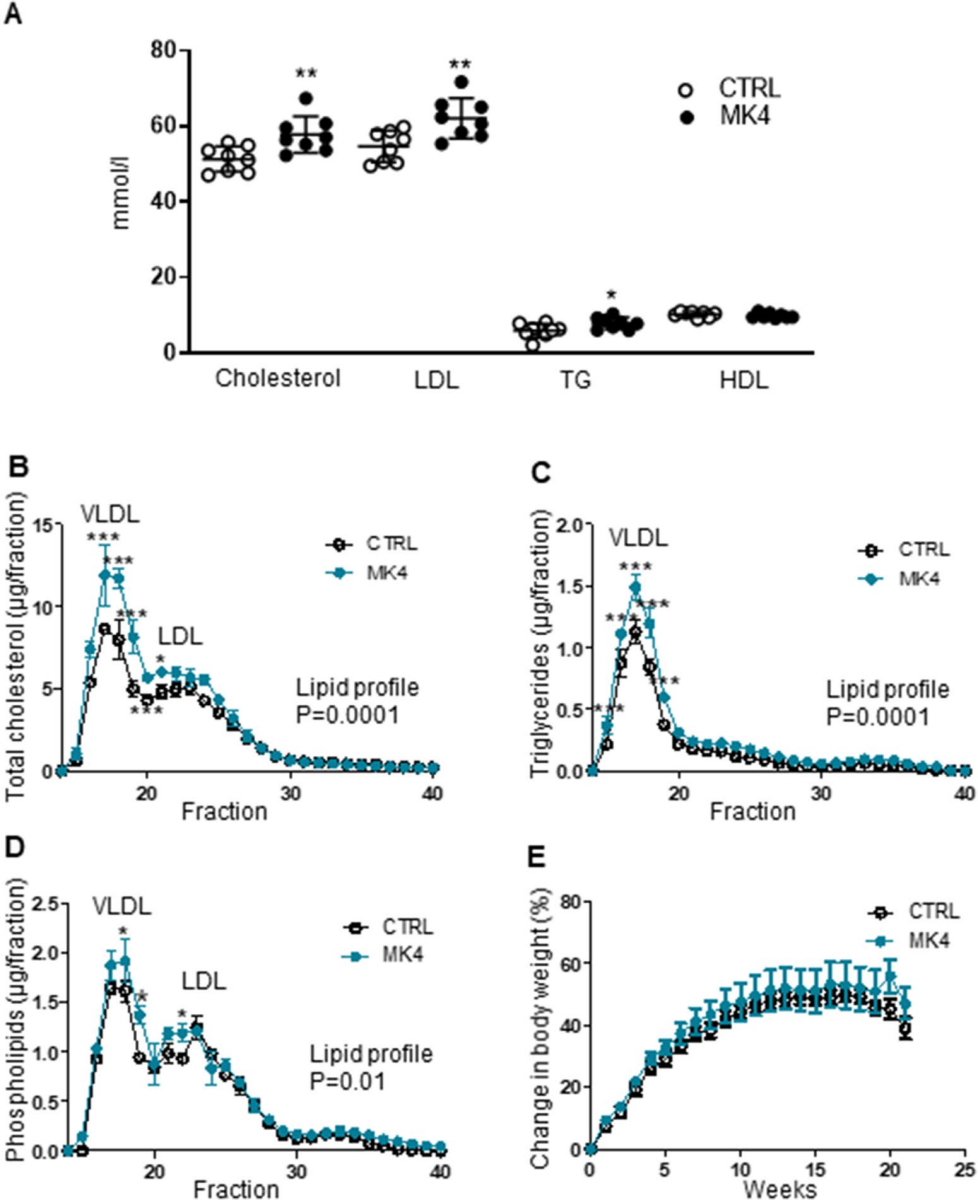
Plasma lipid effects of menaquinone 4 in *LDLr*^*−/−*^*ApoB*^*100/100*^* mice*. (**A**) Plasma lipid levels and (**B**–**D**) lipoprotein profiles. (**E**) Mice body weight gain. LDL, low-density lipoprotein; TG, triglycerides; HDL, high-density lipoprotein; VLDL, very low-density lipoprotein; CTRL (n = 8–9), *LDLr*^*−/−*^*ApoB*^*100/100*^ mice on Western diet (WD); MK4 (n = 8), LDLr^*−/−*^ApoB^100/100^ mice on WD with menaquinone 4. Student´s t-test and 2-way ANOVA with Bonferroni post-hoc test was used for statistical analysis. *p < 0.05, **p < 0.01, ***p < 0.001.

Since WD elevates plasma lipid levels significantly in this genetically modified mouse model, we studied the effect of MK4 on the plasma lipid concentration and lipoprotein profiles. Total cholesterol (+ 13%, *p* < 0.01), triglycerides (+ 33%, *p* < 0.05) and LDL (+ 14%, *p* < 0.01) levels were significantly elevated whereas the HDL fraction remained unchanged in the MK4 treated mice when compared to the control group (Fig. 2A). Plasma lipoprotein profiles were further studied using the FPLC approach and this confirmed the disturbed lipid profile in MK4 treated *LDLr*^*−/−*^*ApoB*^*100/100*^ mice. The main fraction responsible for the elevated total cholesterol and triglycerides was VLDL, i.e. its proportion was markedly increased in MK4 treated mice (Fig. 2B–D). Interestingly, VLDL particles were enriched in all three main lipid classes, i.e. cholesterol (Fig. 2B), triglycerides (Fig. 2C), and phospholipids (Fig. 2D) after MK4 treatment when compared to the controls. In addition, LDL particles were enriched with cholesterol (Fig. 2B) and phospholipids (Fig. 2D) in the MK4 group. The body weight gain was followed weekly and it was similar in both study groups throughout the study (Fig. 2E).

### MK4 increases the expression of lipid metabolism regulating genes

**Figure 3:**
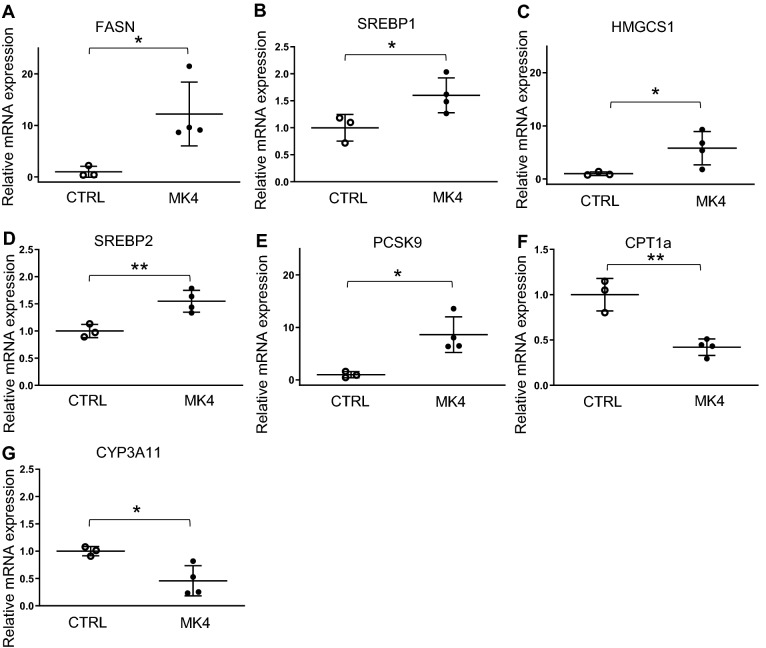
Effect of menaquinone 4 on hepatic gene expression in *LDLr*^*−/−*^*ApoB*^*100/100*^ mice. (**A**) Fatty acid synthase (*Fasn*), (**B**) sterol regulatory element binding transcription factor (*Srebp1*), (**C**) 3-hydroxy-3-methylglutaryl-Coenzyme A synthase 1 (*Hmgcs1*), (**D**) *Srebp22*, (**E**) proprotein convertase subtilisin/kexin type 9 (*Pcsk9*), carnitine palmitoyltransferase 1a (*Cpt1a*) and cytochrome P450 family 3 subfamily A polypeptide 11 (*Cyp3a11*). CTRL (n = 3), *LDLr*^*−/−*^*ApoB*^*100/100*^ mice on Western diet (WD); MK4 (n = 4), *LDLr*^*−/−*^*ApoB*^*100/100*^ mice on WD with menaquinone 4. Student´s t-test was used for statistical analysis. *p < 0.05, **p < 0.01.

To study the molecular mechanisms underlying elevated plasma lipid levels, we analyzed the expression levels of inflammatory and lipid metabolism genes in liver after 18 h exposure of MK4, the expected time when changes in gene expression would be observed. We found that MK4 upregulated the mRNA expressions of triglyceride synthesis genes fatty acid synthase (*Fasn*,12.2-fold, *p* < 0.05) and sterol regulatory element binding transcription factor 1c (*Srebp1c*, 1.6-fold, *p* < 0.05), cholesterol synthesis genes 3-hydroxy-3-methylglutaryl-Coenzyme A synthase 1 (*Hmgcs1*, 5.8-fold, *p* < 0.05), sterol regulatory element binding, factor 2 (*Srebp2*, 1.6-fold, *p* < 0.01), and also proprotein convertase subtilisin/kexin type 9 (*Pcsk9*, 8.6-fold, *p* < 0.05), a protein known to be involved in the regulation of LDL receptor degradation (Fig. 3A–E and Supplementary Table 1). Interestingly, MK4 caused reductions in mRNA expressions of the β-oxidation regulatory gene, carnitine palmitoyltransferase 1a (*Cpt1a*, 0.4-fold, P < 0.01) and cholesterol synthesis related gene cytochrome P450 family 3 subfamily A polypeptide 11 (*Cyp3a11*, 0.5-fold, *p* < 0.05) (Fig. 3F–G and supplementary table 1).

### MK4 diet enhances steatosis and the expression of fibrosis markers in the liver but decreases inflammatory cytokine secretion in the liver

**Figure 4:**
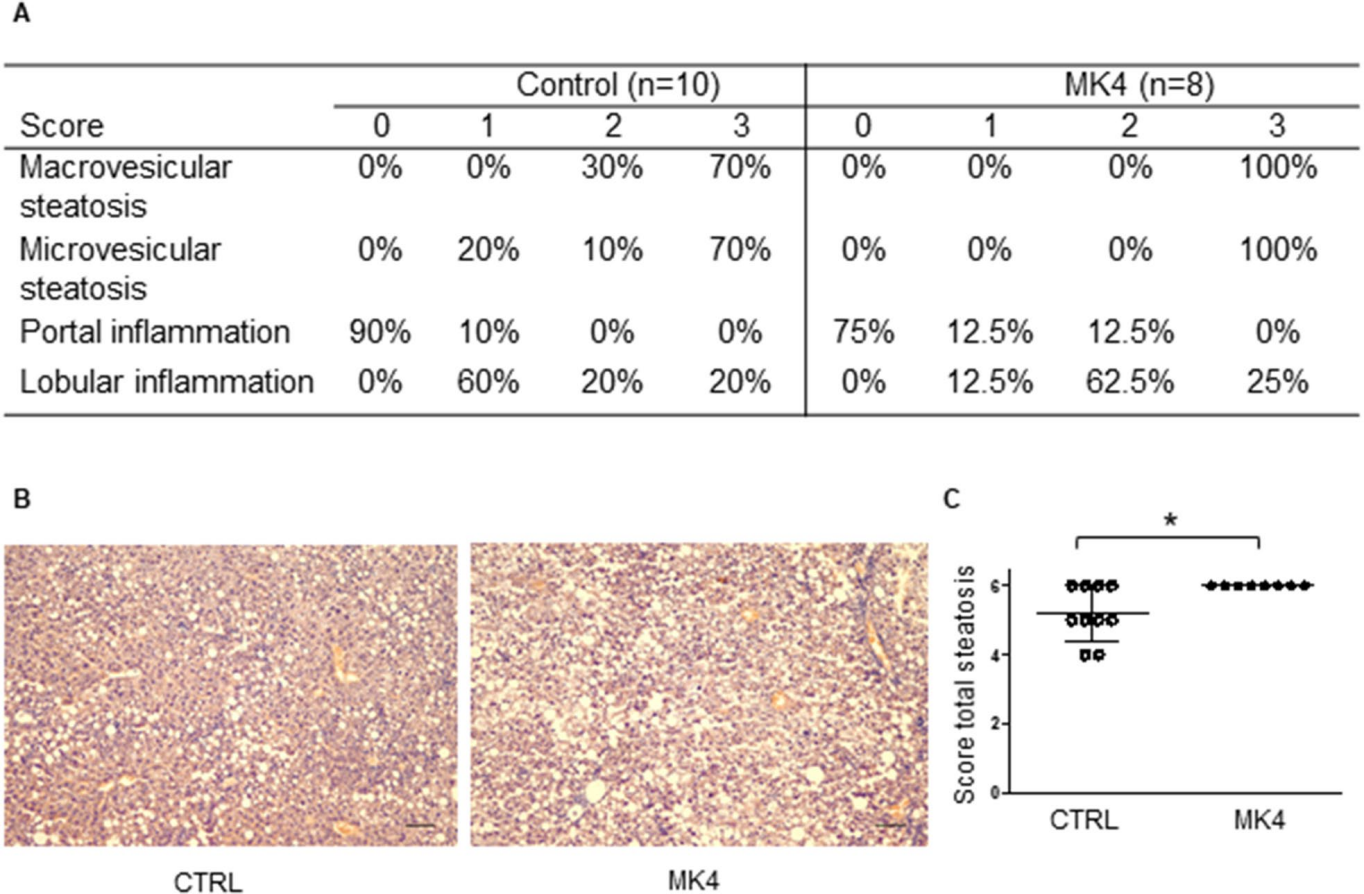
Hepatic effects of menaquinone 4 in *LDLr*^*−/−*^*ApoB*^*100/100*^ mice. (**A**) Steatosis scoring of mice livers. Score 0: negative, score 1: mild, score 2: moderate, score 3: strong. (**B**) Representative images of liver histology with hematoxylin–eosin staining. (**C**) Combined score of micro- and macrovesicular steatosis. Scale bars are 200 µm. CTRL (*LDLr*^*−/−*^*ApoB*^*100/100*^ mice on Western diet (WD); MK4, LDLr^*−/−*^ApoB^100/100^ mice on WD with menaquinone 4. Mann–Whitney U-test was used for statistical analysis. *p < 0.05.

We assessed whether the higher lipid levels induced by the MK4 diet had affected liver lipid accumulation or evoked hepatic steatosis. Histologically, micro- and macrovesicular steatosis and inflammation was detected from the mice livers (Fig. 4A). Indeed, liver tissue exhibited enhanced total steatosis (*p* < 0.05) in the MK4 diet group when compared to the control group (Fig. 4A–C) as assessed by histological scoring of micro- and macrovesicular steatosis. Microvesicular steatosis was located mainly in the centrilobular region and macrovesicular steatosis was identified surrounding the portal areas.

**Figure 5:**
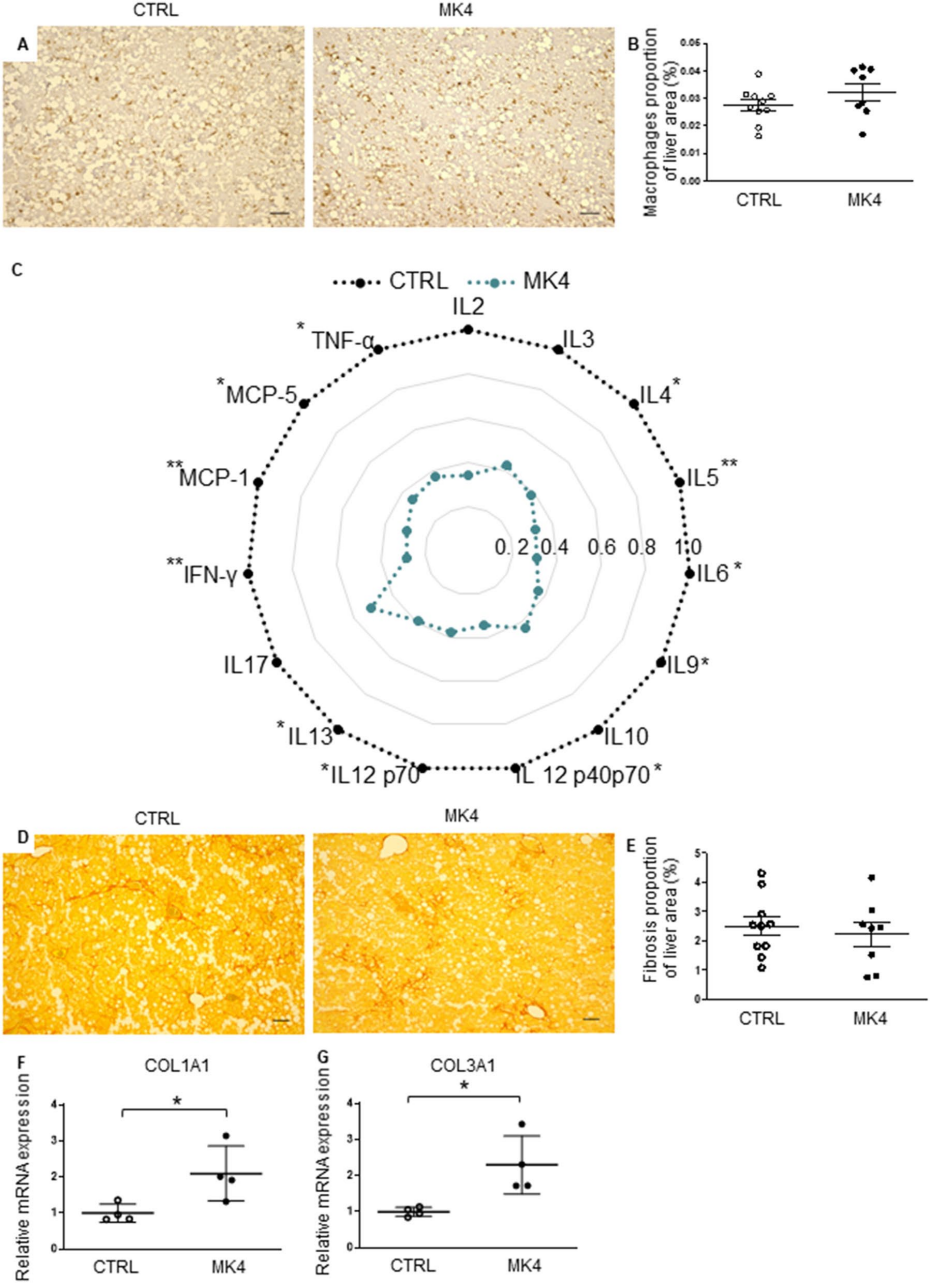
Hepatic effects of menaquinone 4 in *LDLr*^*−/−*^*ApoB*^*100/100*^ mice. (**A**) Representative images of liver macrophages determined by immunohistochemistry of CD68^+^ and (**B**) quantification of macrophages in the liver in CTRL (n = 10) and MK4 (n = 8) mice. (**C**) Cytokine array was done from hepatic tissue of control (n = 6) and MK4 (n = 6) mice. Quantification of the relative expression levels of selected cytokines and chemokines (**D**) Picro sirius red staining for fibrosis and (**E**) quantification of fibrosis in CTRL (n = 10) and MK4 (n = 8) mice. Gene expression of collagens (**F**) *(Col)1a1* and (G) *Col3a1* in CTRL (n = 4) and MK4 (n = 4) mice. CTRL, LDLr^*−/−*^ApoB^100/100^ mice on Western diet (WD); MK4, *LDLr*^*−/−*^*ApoB*^*100/100*^ mice on WD with menaquinone 4. IL, interleukin; IFN-γ, interferon gamma; MCP, monocyte chemoattractant protein, TNFα, tumour necrosis factor alpha. Student´s t-test was used for statistical analysis. *p < 0.05, **p < 0.01. Student´s t-test was used for statistical analysis. Scale bars are 200 µm. *p* < 0.05.

In addition, a sparse degree of lobular inflammation was evident in the liver sections from all of the mice (Fig. 4A). To evaluate the activation of hepatic macrophages, we performed immunohistological staining against CD68^+^ macrophage antibody (Fig. 5A). The control and MK4 group exhibited a similar proportion of the macrophages in the liver (Fig. 5B). However, as vitamin K has been shown to have anti-inflammatory effects, we performed a hepatic cytokine array. We detected a decline in the expression of several central inflammatory cytokines after MK4 treatment when compared to the control group (Fig. 5C). Interestingly, MK4 decreased the expression of both anti-inflammatory cytokines, e.g. interleukin 4 (IL-4) (−62%, *p* < 0.05), IL-5 (−69%, *p* < 0.01), IL-9 (−69%, *p* < 0.05), and pro-inflammatory cytokines e.g. IL-6 (−64%, *p* < 0.05), IL-12p40 (−66%, *p* < 0.05), IL-12p70 (−62%, *p* < 0.05) and IL-13 (−61%, *p* < 0.05). In addition, the protein levels of tumour necrosis factor α (TNFα) (−63%, *p* < 0.05) and interferon-γ (IFN-γ) (−72%, *p* < 0.01) were markedly reduced, as well as those of the chemokines, monocyte chemoattractant protein 1 (MCP1) (−70%, *p* < 0.001) and MCP5 (−65%, *p* < 0.05). No significant changes were detected in protein levels of IL-2, IL-3, IL-10, or IL-17.

Next, fibrosis was identified with Picro Sirius red staining (Fig. 5D). Fibrosis was detected in all of the mouse livers but the proportion of hepatic fibrosis was similar in both study groups (Fig. 5E). Finally, we determined mRNA expression of collagens (COL) 1A1 and 3A1 in the liver. Indeed, the mRNA expression of COL1A1 (2.1- fold, *p* < 0.05) and COL3A1 (2.3-fold, *p* < 0.05) were significantly increased in the MK4 group when compared to the control group (Fig. 5F–G). However, this did not translate into an actual elevation of hepatic fibrosis in the Picro Sirius red analysis.

## Discussion

The present study shows that MK4 exacerbated the lipid profile without affecting the aortic valve morphology in *LDLr*^*−/−*^*ApoB*^*100/100*^ mice. MK4 increased plasma levels of total cholesterol, triglycerides, and LDL levels.

We and others have previously shown that if *LDLr*^*−/−*^*ApoB*^*100/100*^ mice consume a WD for 5 months this mimics the early stage of CAVD^8,10^, and importantly, this is the point during disease progression that a drug treatment is likely to be efficacious. In our study, MK4 did not affect valve area or the proportion of calcification nor fibrosis in the aortic valves. In addition, MK4 did not affect atheroma plaque calcification or plasma ALP levels. In previous studies, both vitamin K1 and K2 have been shown to exert positive effects on vascular calcification^6,11^ and the progression of aortic valve calcification in patients with asymptomatic or mildly symptomatic CAVD^7^; however, there exist also contradictory findings. MK4 had no beneficial effect on the coronary artery calcification score in patients with at least one coronary risk factor^12^ and MK7 tended to increase active calcification in type 2 diabetic patients with cardiovascular disease^13^. Similarly, MK7 had no effect on aortic calcification in haemodialytic chronic kidney disease patients or in uremic Sprague–Dawley rats^14,15^. Furthermore, MK-4 was reported to accelerate aortic valve calcification in cultured human aortic valve interstitial cells^16^.

In the present study, MK4 increased the proatherogenic plasma lipid and lipoprotein profile. In humans, several studies have reported a positive association between vitamin K1 and plasma cholesterol and triglyceride levels^17,18^. In addition, Kamali et al.^19^ found that vitamin K fluctuated in a similar manner as triglycerides during the circadian rhythm. Previously, some work done in experimental animals has examined how vitamin K can modify lipid metabolism. It has been reported that MK7 did not cause statistically significant differences in the levels of total cholesterol or triglycerides in *ApoE*^*−/−*^*LDLr*^*−/−*^ mice^20^, although there was a trend towards an increase in those of triglycerides. In another study, MK7 reduced plasma total cholesterol levels in uremic Sprague- Dawley rats^15^. Vitamin K1 reduced the body weight gain, total cholesterol, and triglycerides in Swiss albino mice on a high fat diet^21,22^. Kawashima et al.^23^ found that vitamin K1 lowered the total cholesterol but had no effect on the levels of triglycerides or HDL in hypercholesterolemic rabbits. In our study, plasma MK4 levels in control mice were at same level as plasma MK7 levels of the control mice in the study of Lupo et al.^15^. MK4 supplementation clearly increased plasma MK4 levels. Compared to previous studies, we used MK4, adopting a longer duration of treatment (5 months *vs*. the published 8–10 weeks), and a different animal model. *LDLr*^*−/−*^*ApoB*^*100/100*^ mice have a human-like lipoprotein profile and WD causes a marked increase of plasma lipid levels reflecting a hypercholesterolemic phenotype^24,25^.

Higher plasma lipid values could indicate an overall accumulation of lipids in peripheral locations such as vascular wall and aortic valve. In our study, the MK4 group displayed enhanced steatosis compared to the controls but the presence of MK4 did not accelerate valvular thickening. Vitamin K is a lipid soluble vitamin and it is absorbed and carried by lipoproteins and also targeted to be taken up by the liver^26^. We found that MK4 exaggerated the expression of genes required for fatty acid and cholesterol regulatory pathways in the liver by inducing the expressions of *Fasn*, *Hmg*cs and *Pcsk9* as well as their transcriptional regulators, *Srebp1c* and *Srebp2*^27,28^. *Fasn* enhances production of fatty acids, leading to exaggerated triglyceride synthesis^29^ whereas *Hmgcs* synthesizes HMG-CoA which is further translated into cholesterol^30^. *Pcsk9* is capable to increase LDL receptor degradation and increase plasma total cholesterol and LDL levels^31^. Master regulators of lipid homeostasis, *Srebp1* is linked to lipogenesis and *Srebp2* to cholesterol metabolism pathways and it can also activate *Pcsk9*^28,32^. In addition, a decline in *Cyp3a11* and *Cpt1a* gene expression was detected. This is in line with the previous work of Hashimato et al.^33^, demonstrating that knockdown of CYP3a in mice led to an increase of cholesterol biosynthesis via *Srebp2*. *Cpt1a* is a rate limiting for mitochondrial β-oxidation and the substrate of *Fasn,* Malonyl-CoA, is an inhibitor of *Cpt1a*^34^.

All in all, the changes at hepatic gene expression level are in concordance with the increased plasma levels of triglycerides and total cholesterol. The only previous study investigating the effect of vitamin K on hepatic cholesterol metabolism showed that MK7 increased the gene expression of *LDLr* and *Hmgcr* and reduced the expression of *Pcsk9* in human hepatic cancer cell line^15^. The comparison of these results to ours is limited by the use of MK4 and non-oncogenic hepatic tissue of *LDLr* deficient mice in our study.

The degrees of lobular inflammation, macrophage accumulation and fibrosis were similar in both study groups despite the increased liver steatosis. However, we found enhanced mRNA expression of collagens *Col1a1* and *Col3a1* in liver, which may precede increased fibrosis that was still not detectable after 5-months of the MK4 diet. We also found that MK4 decreased the levels of several inflammatory markers in the liver, including IL-6, MCP-1 and TNFα. This may be explained by the anti-inflammatory effects of MK4. It has been reported that MK4 can reduce the IL-6, IL1β and TNFα mRNA levels in lipopolysaccharide-induced mouse RAW264.7 and microglia-derived cells^35,36^. Similarly, in the Framingham offspring study, excess vitamin K attenuated the general plasma inflammatory index as well as lowering the level of TNFα^37^. As the overall expression of both pro- and anti-inflammatory cytokines and chemokines decreased in our study, these findings may indicate that MK4 directly suppresses the hepatic nuclear factor kappa B (NF-κB) pathway which is in line with previous reports^21,22,35,36^. Thus, we propose that MK4 might have a dual role in lipid metabolism and inflammation by diminishing inflammatory cytokine secretion and promoting the gene expression of lipid metabolism regulating genes in liver.

Hypercholesterolemic *LDLr*^*−/−*^*ApoB*^*100/100*^ mice are not only a model of CAVD but also a relevant model for human FH. Most (90%) FH patients have a mutation in the LDLr gene, whereas only a minority of patients have mutations in the apolipoprotein-B or PCSK9 genes that are associated with high cholesterol levels already in utero^38^. Since FH patients have a higher risk of developing CAVD, our study suggests that FH patients might be more vulnerable to the hyperlipidemic side effects of vitamin K. In our study, we used MK4, which is an active metabolite of vitamin K1 and is present in meat, cheese and eggs^26^. The European Union Scientific committee of food concluded that it is not necessary to set upper intake level for vitamin K as vitamin K has not been observed to evoke side effects, even at a high dose level^39^. However, the possible susceptibility of FH patients to the unfavourable side effects of this vitamin should be evaluated carefully in the future, since vitamin K could influence the CVD risk by increasing further the poor proatherogenic lipid profile in certain patient groups.

In this study, we used the transgenic mouse model and a high fat diet to mimic human CAVD and FH. In mice, most of the cholesterol is carried in HDL particles, whereas in humans in LDL and VLDL particles. Thereby, elevated plasma LDL and VLDL levels must be generated via transgenic mouse models or/and feed with rich fat and cholesterol content diet^24^. Consequently, extrapolation of the results to humans may not be straightforward and further studies are required to determine the effect of vitamin K on plasma lipids in patients with FH.

Here, we demonstrate that MK4 caused a deranged plasma lipid profile without affecting the aortic valve morphology in *LDLr*^*−/−*^*ApoB*^*100/100*^ mice. The mice consuming the MK4 diet had increased plasma levels of LDL and triglycerides, hepatic steatosis, and mRNA expression of hepatic genes required for triglyceride and cholesterol metabolism. Consequently, MK4 may have non-beneficial effects on lipid levels, especially in the presence of hypercholesterolemia.

## Materials and methods

### Animals

Male *LDLr*^*−/−*^*ApoB*^*100/100*^ mice (C57BlJx129Sv, Jackson laboratories) were randomly divided into the Western-type diet (WD) (n = 13) and the MK4 (n = 14) groups. At the age of 3 months, mice were fed with the WD (Harlan Teklad 88137, containing 42% of energy from fat) or WD with additional 0.2 mg/g of MK4 (V9378, Sigma-Aldrich, USA) for 5 months. The diets were prepared as follows. WD pellets were ground up and MK4 was added to the powdered pellets according to pharmaceutical principles using a geometric series. The powder of WD was kept in ice throughout the process. The WD for control mice was processed similarly without the incorporation of MK4.

At the end of the study, the mice were fasted for 6 h and euthanized with CO_2_ and cervical dislocation, blood, heart and liver samples were collected. In the gene expression studies, the mice were euthanized at 18 h after the start of the MK4 diet. The mice were housed in individual cages with free access to tap water in a room with a controlled humidity and a temperature. A 12 h light and 12 h dark environmental light cycle was maintained. The experimental design was approved by the Animal Ethics Committee of The State Provincial Office of Southern Finland, decision number ESAVI/11642/04.10.07/2014. All the experiments conformed to the guidelines from Directive 2010/63/EU of the European Parliament on the protection of animals used for scientific purposes and by the ARRIVE guidelines and recommendations of NC3Rs.

### MK4 plasma levels analysis

MK4 Plasma levels were determined at Admescope Ltd, in Oulu. The samples were prepared by protein precipitation (ratio 1:2) using acetonitrile containing of 1% of formic acid. The samples were then analysed with a UPLC-HRMS (Waters Acquity UPLC + Thermo Q-Exactive Focus Orbitrap MS) using a Waters BEH C8 (1.7 µm particle size, 50 × 2.1 mm) UPLC column with Methanol and 0.1% acetic acid as mobile phases for the chromatography and an APCI + ionization at the mass-spectrometer. The samples were quantified against standard samples prepared into mouse plasma by spiking blank matrix into concentrations ranging from 2 to 5000 ng/ml of analyte. Obtained accuracies ranged from 93.3 to 107.9%, with Snedecor precision of 11.7%. Quality control samples at mouse plasma concentrations of 30, 300 and 3000 ng/ml, were also included in the analysis. The Accuracy of the control samples ranged from 92.6 to 93.2%.

### Histopathology

Tissue samples for histological analysis were taken at the time of sacrifice. Mice were euthanized with CO_2_, perfused with phosphate buffered saline and hearts and livers were collected and fixed in 4% paraformaldehyde and embedded in paraffin. Cross-sections of aortic root, 5 µm thick, were stained with hematoxylin–eosin (HE), Masson trichrome for fibrosis, Alizarin red for calcification and MAC-3 (CD107b, BD Pharmingen, USA) immunohistochemistry for macrophages were used. Valve area was determined from two selections. 100 µm apart from each other and the average of the valve area was calculated. Cross-sections of the liver, 7 µm thick, were stained with HE to detect steatosis and Picro Sirius red to detect collagen. HE sections were scored from 0 to 3 as previously described^40,41^. Immunohistochemical detection of macrophages was performed using CD68 antibody (ab125212, Abcam, UK). In the analysis, the proportions of CD68-positive cells, fibrosis and calcification were calculated (positive area/total area*100%) from one section with Image J analysis software^42^. All analyses were done in a blinded manner.

### Echocardiography

Fujifilm VisualSonics Vevo 2100 (Toronto, Canada) was used for transthoracic echocardiography, which was conducted under isoflurane anesthesia (induction, 3.0% isoflurane—Baxter International, USA, 400 mL air; maintenance, 2.7% isoflurane, 400 mL air—Univentor 400 anesthesia unit, Malta). A long‐axis M‐mode view was adopted when analyzing aortic diameter, and Doppler echocardiography was used in determination of the velocity and the pressure gradient. Vevo2100 software as previously described^8^. Data analysis was done in a blinded fashion.

### Isolation and analysis of RNA

Total RNA was extracted from the liver with TRI-reagent (Sigma-Aldrich, USA). For real-time quantitative polymerase chain reaction (RT-qPCR) analyses, cDNA was synthesized from total RNA with M-MuLV Reverse Transcriptase, (Fermentas/Thermo Scientific, USA), and the RT-qPCR was performed as previously described^43^ using Taqman predesigned Real Time PCR assays (Thermo Fisher Scientific, USA). Assays are listed in the Supplementary Table S1. From each sample, duplicate technical replicates were analysed, and the gene expression was normalized with 18S rRNA measured from the same samples using ΔΔCT method.

### Lipid analysis

Total cholesterol, LDL-cholesterol (LDL-C), high-density lipoprotein (HDL)-cholesterol (HDL-C), and triglycerides with were analyzed with a photometric assay at the MoVet veterinary service laboratory (Kuopio, Finland).

### Fast performance liquid chromatography (FPLC) analysis

Lipoprotein profiles were analyzed by fast performance liquid chromatography (FPLC) as described previously^44^. First, plasma samples were pooled (n = plasma from 5–7 mice/pool). Pooled plasma samples were fractionated using a Merck Hitachi FPLC system equipped with a pump model L-6200A (Merck, Darmstadt, Germany-Hitachi, Tokyo, Japan), a UV detector model L-4200, an integrator model D-7500, and a manual injector with 1.0 mL sample loop. Pool aliquots of 150 μl were applied on a Superose 6 h 10/300 size-exclusion chromatography column (Pharmacia Biotech, Uppsala, Sweden) using phosphate-buffered saline (pH 7.4) as a mobile phase at a flow rate of 0.5 ml/min. The detection wavelength was 280 nm and 0.5 ml fractions were collected using Retriever 500 fraction collector. The fractions were analyzed for cholesterol, triglyceride, and phospholipid concentrations.

### Cytokine array

Mouse cytokine array (ab133993, Abcam, UK) designed to detect 22 cytokines was used following the manufacturer´s instructions. Proteins were extracted from livers of 8-month old mice (n = 6 in both groups) using lysis buffer provided by the array supplemented with protein inhibitors (cOmplete ULTRA tablets, Roche, Switzerland). Protein concentration was determined using BioRad protein assay (BioRad, UK) based on the Bradford method, and 200 µg of total liver protein was used for cytokine determination. Results were analyzed with Image J software as previously described^45^.

### Statistical analysis

The statistical analysis was conducted with unpaired Student´s t-test, Mann–Whitney U-test or repeated measurement 2-way ANOVA with Bonferroni post-hoc correction using GraphPad Prism5 software. The Pearson correlation coefficient method was used in the correlation analysis. A value of *p* < 0.05 was considered statistically significant. Numerical values are shown as mean ± standard deviation (SD) or mean ± 95% confidence intervals.

## Supplementary information

Supplementary Information
