# Supplementary material for: Menaquinone 4 increases plasma lipid levels in hypercholesterolemic mice
Source: Sci Rep. 2021 Feb 4;11:3014. doi: 10.1038/s41598-021-82724-0 (PMC7862223; doi:10.1038/s41598-021-82724-0)
Supplement: Supplementary file 1 — Supplementary Information [file 41598_2021_82724_MOESM1_ESM.pdf]

SUPPLEMANTAL MATERIALS

Title: Menaquinone 4 increases plasma lipid levels in hypercholesterolemic mice

Jonna Weisell[1], Anna-Kaisa Ruotsalainen[2], Juha Näpänkangas[3], Matti Jauhiainen[4], Jaana Rysä[1*]

[1]School of Pharmacy, University of Eastern Finland, Kuopio, Finland

[2] A.I. Virtanen Institute for Molecular Sciences, University of Eastern Finland, Kuopio, Finland

[3] Department of Pathology, University of Oulu, Oulu University Hospital, Oulu, Finland

[4] Minerva Foundation Institute for Medical Research, Helsinki, Finland

* Corresponding author

SUPPLEMENTAL TABLES

Supplementary table 1. Assay targets used in the hepatic gene expression analysis and results of hepatic gene expression analysis. Red colour indicates statistical significance at the level of P<0.05.

Gene	Gene symbol	Assay number	Fold-change* (P-value)
3-hydroxy-3-methylglutaryl-Coenzyme A reductase	HMGCR	Mm01282499m1	2.7 (0.08)
3-hydroxy-3-methylglutaryl-Coenzyme A synthase 1	HMGCS1	Mm01304569m1	5.8 (0.049)
Acetyl-Coenzyme A acetyltransferase 1	ACAT1	Mm00507463m1	0.9 (0.71)
ATP binding cassette subfamily B member 1a	ABCB1a	Mm00440761m1	0.6 (0.10)
ATP binding cassette subfamily B member 1b	ABCB1b	Mm00440736m1	1.1 (0.47)
ATP synthase, H+ transporting, mitochondrial F0 complex, subunit C1 (subunit 9)	ATP5G1	Mm01307015g1	1.2 (0.44)
ATP-binding cassette, sub-family A (ABC1), member 1	ABCA1	Mm00442646m1	1.0 (0.89)
ATP-binding cassette, sub-family G, member 1	ABCG1	Mm00437390m1	0.8 (0.50)
Carnitine palmitotyltransferase 1a, liver	CPT1A	Mm01231183m1	0.4 (0.002)
Collagen Type I Alpha 1 Chain	COL1A1	Mm00801666g1	2.1 (0.03)
Collagen Type III Alpha 1 Chain	COL3A1	Mm01254476m1	2.3 (0.02)
Cytochrome P450 family 3 subfamily A polypeptide 11	CYP 3A11	Mm00731567m1	0.5 (0.02)
Cytochrome P450, family 7, subfamily a, polypeptide 1	CYP7A1	Mm00484150m1	0.5 (0.22)
Farnesyl diphosphate farnesyl transferase 1	FDFT1	Mm01598574g1	1.7 (0.17)
Fatty acid synthase	FASN	Mm00662319m1	12.2 (0.03)
LDL receptor related protein 1	LRP1	Mm00464608m1	0.7 (0.10)
Lipoprotein Lipase	LPL	Mm00434764m1	0.6 (0.08)
Matrix Gla Protein	MGP	Mm00485009m1	0.6 (0.13)
Microsomal Triglyceride Transfer Protein	MTTP	Mm00435015m1	1.1 (0.83)
Proprotein convertase subtilisin/kexin type 9	PCSK9	Mm01263610m1	8.6 (0.01)
Ribosomal 18s	18s	Hs99999901s1	-
Scavenger receptor class B, member 1	SCARB1	Mm00450234m1	0.8 (0.15)
Scavenger Receptor Class B, Member 3	CD36	Mm00432403m1	0.5 (0.08)
Secreted phosphoprotein 1	SPP1	Mm00436767m1	1.3 (0.77)
Sterol regulatory element binding transcription factor 1	SREBP1C	Mm00550338m1	1.6 (0.045)
Sterol regulatory element binding, factor 2	SREBP2	Mm01306292m1	1.5 (0.01)
Tumor necrosis factor receptor superfamily member 11b	TNFRSF11b	Mm00435454m1	1.0 (0.89)

* Relative expression of MK4 group to control group

SUPPLEMENTAL FIGURES

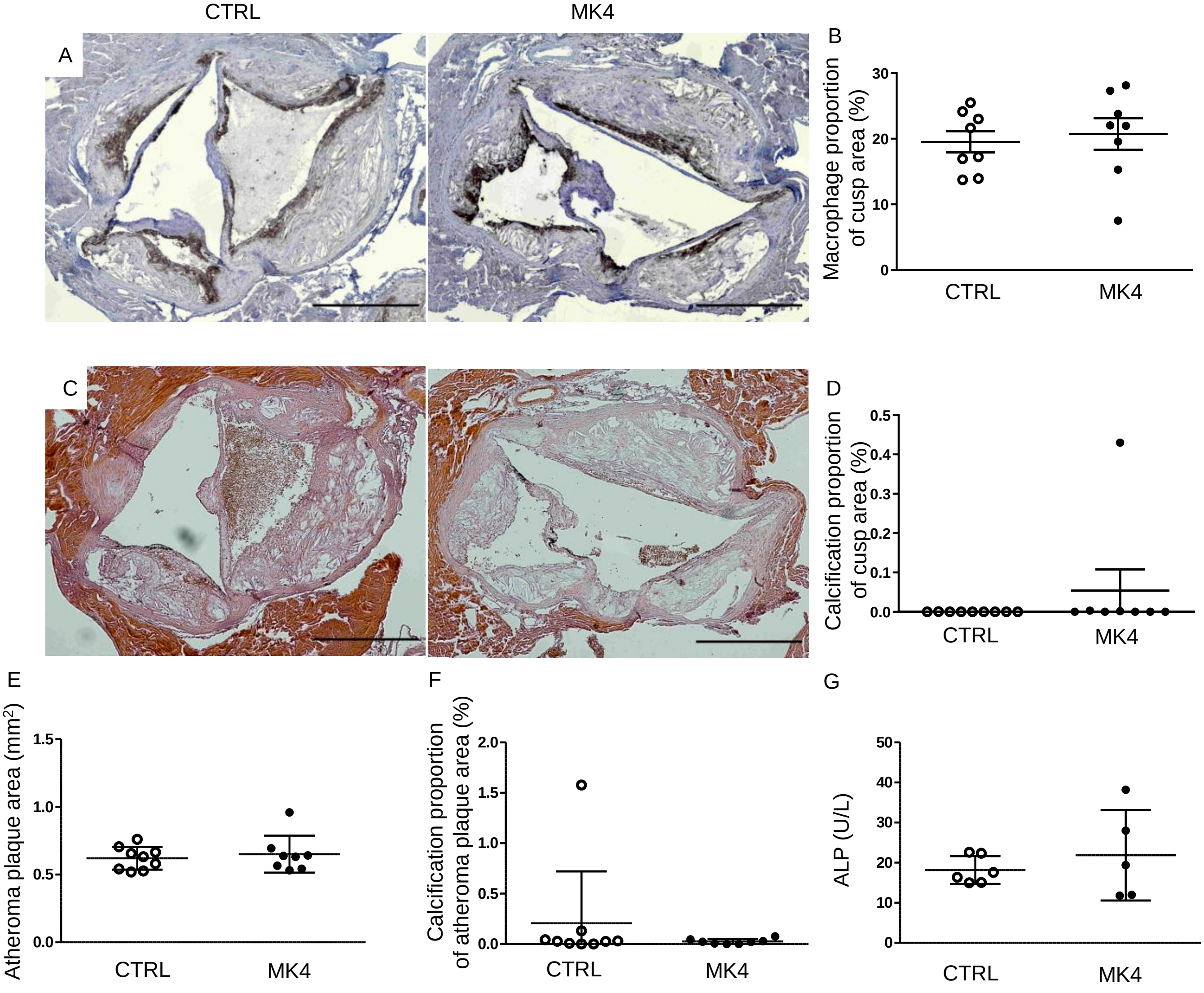

Supplemental Figure 1. Effect of menaquinone 4 on aortic valve morphology. Representative images of A) macrophage staining MAC-3 and B) quantification of macrophages. C) Alizarin red staining for calcification. D) Quantification of calcification in the cusp area. E) Atheroma plaque area and F) proportion of calcification in the atheroma plaque area. G) Alkaline phosphatase (ALP) plasma levels. Scale bars 500µm. CTRL (n=8-9), LDLr$^{-/-}$ApoB$^{100/100}$ mice on western diet (WD); MK4 (n=8), LDLr$^{-/-}$ApoB$^{100/100}$ mice on WD with menaquinone 4. Student t-test and Mann-Whitney U-test was used for statistical analysis.

A

Pos	Pos	Neg	Neg	GCSF	GM-CSF	IL-2	IL-3
Pos	Pos	Neg	Neg	GCSF	GM-CSF	IL-2	IL-3
IL-4	IL-5	IL-6	IL-9	IL-10	IL-12 p40p70	IL-12 p70	IL-13
IL-4	IL-5	IL-6	IL-9	IL-10	IL-12 p40p70	IL-12 p70	IL-13
IL-17	IFN-γ	MCP-1	MCP-5	RANTES	SCF	sTNFRI	TNFα
IL-17	IFN-γ	MCP-1	MCP-5	RANTES	SCF	sTNFRI	TNFα
Thromb opoietin	VEGF	Blank	Blank	Blank	Blank	Blank	Pos
Thromb opoietin	VEGF	Blank	Blank	Blank	Blank	Blank	Pos

Cytokine array

B

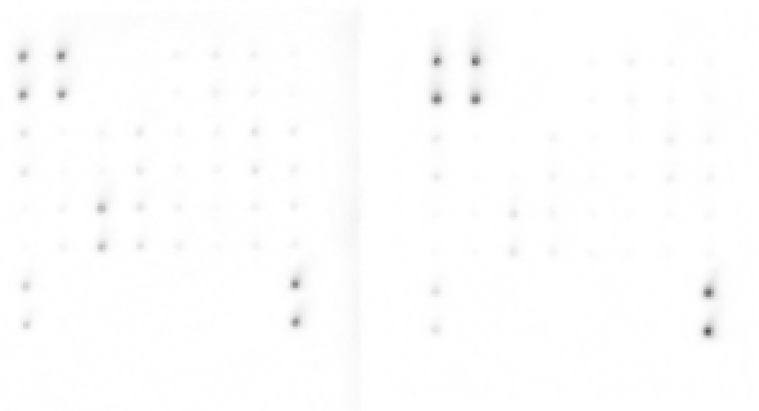

CTRL MK4

Supplemental Figure 2. A) A schematic representation of the cytokine/chemokine spot positions in duplicate on the membrane with respective internal controls. B) Representative images of control and MK4 membranes. CTRL, LDLr$^{-/-}$ApoB$^{100/100}$ mice on western diet (WD); MK4, LDLr$^{-/-}$ApoB$^{100/100}$ mice on WD with menaquinone 4.
